# Determination of the length of single-walled carbon nanotubes by scanning electron microscopy

**DOI:** 10.1016/j.mex.2018.11.004

**Published:** 2018-11-07

**Authors:** Stefania Sandoval, Magdalena Kierkowicz, Elzbieta Pach, Belén Ballesteros, Gerard Tobias

**Affiliations:** a Institut de Ciència de Materials de Barcelona (ICMAB-CSIC), 08193, Bellaterra, Barcelona, Spain; b Catalan Institute of Nanoscience and Nanotechnology (ICN2), CSIC and The Barcelona Institute of Science and Technology, Campus UAB, Bellaterra, 08193, Barcelona, Spain

**Keywords:** Length determination of carbon nanotubes, Dispersion, Length distribution, Shortening, Cutting, Atomic force microscopy

## Abstract

A methodology is presented to determine the length of well individualized single-walled carbon nanotubes (SWCNTs) by means of scanning electron microscopy (SEM). Accurate measurements on wide areas of the sample can be achieved in an easy, fast and trustworthy manner. We have tested several supports and solvents to optimize the dispersion of SWCNTs, as well as the SEM imaging conditions. The optimal methodology goes via dispersion of the sample in ortho-dichlorobenzene, deposition onto a continuous carbon film supported on a copper TEM grid, and SEM imaging at 2 kV in secondary electrons mode using a through-in-lens detector.

• Individualization of SWCNTs is achieved by dispersion of SWCNTs in ortho-dichlorobenzene and deposition onto TEM grids
• Individual SWCNTs are imaged by SEM
• Length determination by SEM is as precise as AFM

## Graphical abstract

**Figure:**
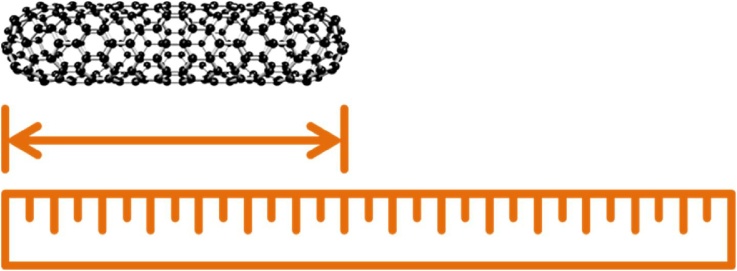

Specifications Table

**Table:** 

Subject area	• Chemistry • Immunology and Microbiology • Materials Science • Pharmacology, Toxicology and Pharmaceutical Science • Physics and Astronomy
More specific subject area	Nanoscience and Nanotechnology
Method name	Length determination of carbon nanotubes
Name and reference of original method	Kierkowicz M, Pach E, Santidrián A, Sandoval S, Tobías-Rossell E, Kalbáč M, et al. Comparative study of shortening and cutting strategies of single-walled and multi-walled carbon nanotubes assessed by scanning electron microscopy. Carbon, 139 (2018), pp. 922–932

## Method details

### Overview

Carbon nanotubes (CNTs) combine a myriad of excellent properties and are being explored for a wide variety of applications ranging from composite materials to the biomedical field, going through electronic devices. They are characterized by a large aspect ratio and hollow core which allows both their exohedral [1,2] and endohedral [[3], [4], [5], [6]] functionalization, further expanding their range of application. CNTs of different lengths are desired depending on the targeted applications. For instance, whereas long CNTs are more suitable for the production of carbon nanoyarns [7], short CNTs are desired for drug delivery and biomedical imaging [1,8]. Short CNTs not only present a higher biocompatibility [9] but can also enhance their performance as contrast agents [10]. As-produced CNTs typically have lengths in the micrometer range, therefore a variety of shortening and cutting strategies have been developed to modulate the length distribution of the as-produced materials. These include oxidative chemical treatments [11,12], mechanical grinding [13], lithography [14], sonication [15], electron-beam [16] and continuous laser cutting [17], which have been described in literature for CNTs shortening. When the cutting process results in CNT lengths of 20–100 nm these have been defined as ultrashort carbon nanotubes (US-tubes) [18].

A fast and efficient characterization tool is necessary to assess the length distribution of both as-produced and processed CNTs. In this area, microscopy techniques are taking the lead. Both, atomic force microscopy (AFM) [11,19] and transmission electron microscopy (TEM) [19,20] are the most usual techniques employed for this purpose. However, the acquisition of images of large areas containing the sample is time consuming, especially in AFM, which represents an important drawback at the time of providing statistically representative data of CNT length.

The use of scanning electron microscopy (SEM) for this purpose arises as an interesting alternative to determine the length distribution of CNTs. SEM is in general more accessible than AFM and TEM and provides accurate measurements for wide areas of the sample in an easy, fast and trustworthy manner. Moreover, most SEM samples require minimal preparation. A previous plasma cleaning can be performed to the sample to prevent contamination during SEM analysis [21].

The present methodology has been recently employed in a comparative study of shortening and cutting strategies of single-walled and multi-walled carbon nanotubes [22].

### Selection of solvent, support and imaging conditions

A major problem to determine the length distribution of SWCNTs is that these nanomaterials are usually present in the form of aggregates due to Van der Waals interactions [23]. In contrast, MWCNTs processing is easier and nanotubes separate much better. CNTs debundling can be achieved by dispersion in a suitable organic solvent, in aqueous solutions using surfactants or by surface modification with functional groups [1,2]. However, the complete individualization of SWCNTs while preserving their structure remains a challenge. In the present study we have focused on SWCNTs since this material is more difficult to disperse and to image by SEM. Hence, the results obtained can be easily translated to samples of MWCNTs. As mentioned, SEM analysis of length distribution has been recently employed on a comparative study of shortening and cutting strategies for both SWCNTs and MWCNTs [22]. CNTs from different sources (chemical vapor deposition and arc-discharge) were employed in this study. Furthermore, since different strategies were employed to shorten the length of CNTs, some of the analyzed CNTs had functional groups attached to their structure. Thus, SEM can be employed to analyze the length distribution of both pristine and functionalized CNTs from different sources.

We tested several supports and solvents to optimize the dispersion of CNTs, which are summarized in Table 1. Various techniques were used in order to disperse SWCNTs: sonication, centrifugation, spin coating and combined approaches. Next, the dispersed samples were dropcasted onto the chosen supports (silicon wafer, mica, alumina stub, TEM grid), or filtered through membranes (polycarbonate, teflon) that would act as a support. Then they were analyzed by SEM. Based on the obtained results we selected the best protocol for the determination of the length distribution of CNT samples, which is described in the next section.

**Table 1:** Summary of supports, solvents and protocols tested for dispersion of CNTs.

**Fig. 1:**
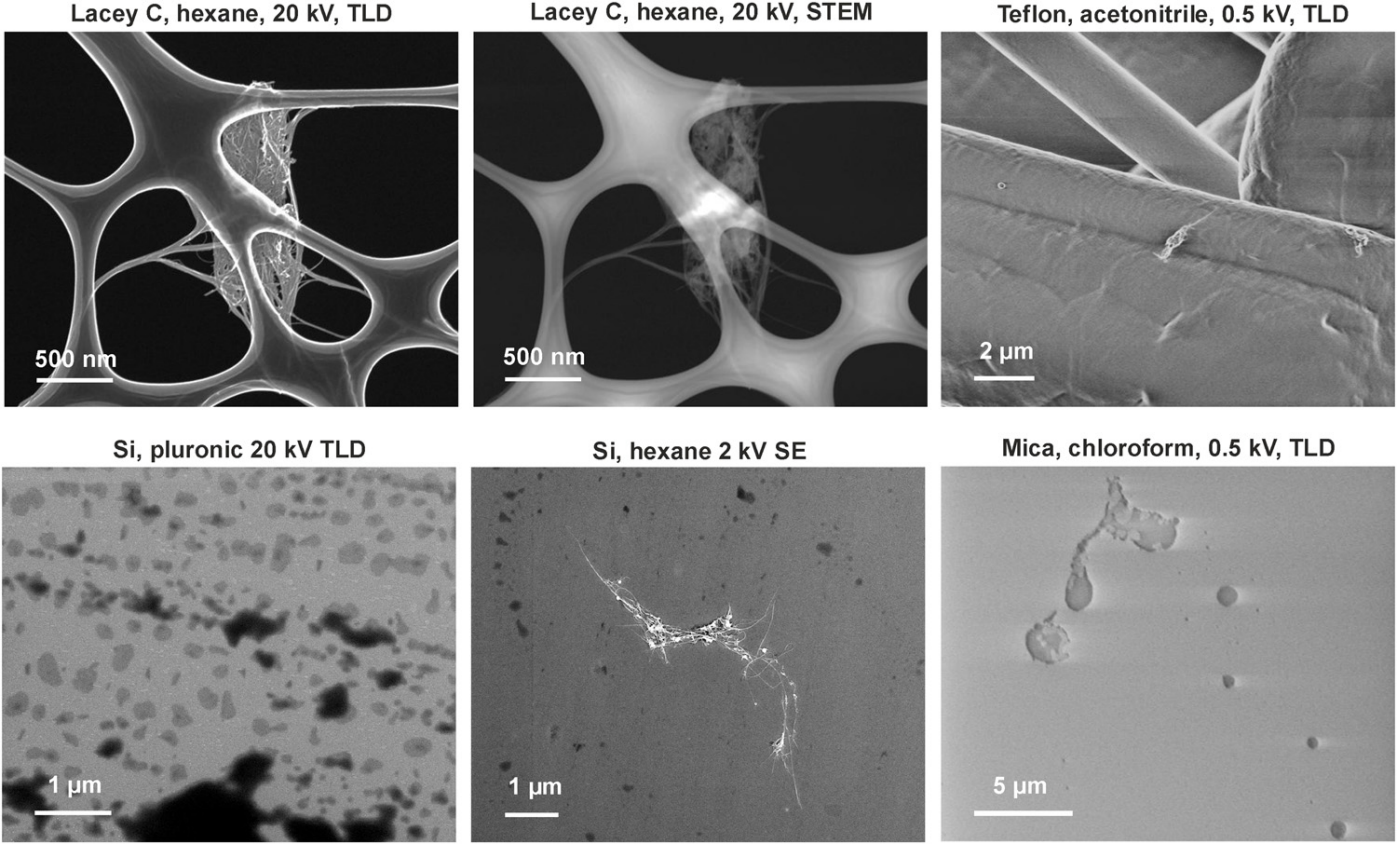
SEM images of steam treated SWCNTs acquired for the selection of solvent, support and imaging conditions.

Fig. 1 shows SEM images after testing different processing and imaging conditions. During the process of searching for a suitable support, TEM grids with lacey /holey carbon film were employed. As it can be seen in the images, the holes in both films are relatively large (∼1 μm) and therefore short nanotubes could easily pass through them and hence bring false results. Teflon was found to be a quite irregular surface so the small nanotubes were difficult to be distinguished from the support. In case of mica, the nonconductive characteristics of the material that charges under an electron beam irradiation even at low voltages, make difficult the acquisition of images. Imaging of SWCNTs on mica under minimal charging conditions (500 V) leads to lower resolution images and hence low precision visualization. A similar drawback was found when using polycarbonate membranes, which undergo charging even at 200 V when imaged with the through-the-lens secondary electron detector (TLD). In backscattered electrons (BSE) mode at 500 V the image seemed to improve but the nanotubes did not appear with enough contrast. Additionally, lower resolution was observed in BSE mode when compared with secondary electrons (SE) mode (used with TLD detector).

Another tested support was a silicon wafer, which seemed to be a good option due to its smooth surface and a good contrast with carbon nanotubes. However, it was found that when depositing a solution of SWCNTs on top of it, the sample formed aggregates during the evaporation of the solvent. Silicon wafers can for instance be useful for SEM analysis of SWCNT networks [24].

All of the abovementioned problems emerging from support material were overcome when SWCNTs were deposited on continuous carbon film covering TEM grid. Its surface provided a smooth face, which offered a good contrast with carbon nanotubes in SE mode. Furthermore, high permeability of the film permitted the solvent to instantaneously pass through it, allowing deposition of well dispersed individual carbon nanotubes when drop-casted from a solution.

**Fig. 2:**
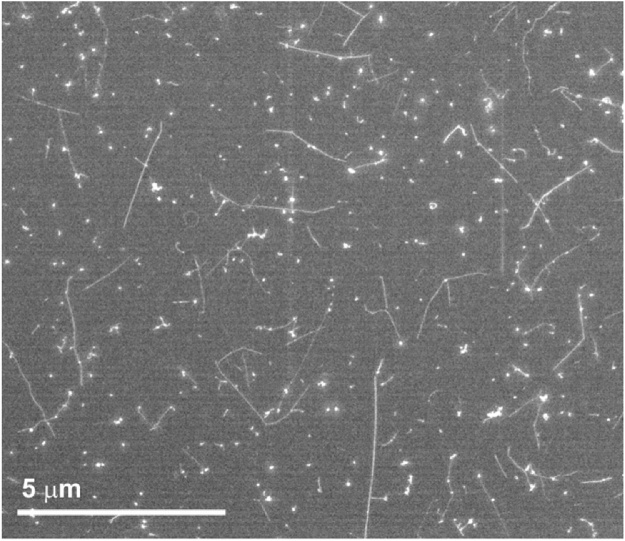
Low magnification SEM image of SWCNTs dispersed in ortho-dichlorobenzene and deposited onto a continuous carbon film grid. The bright dots are attributed to catalytic and graphitic particles present in the as-received material.

Regarding the solvents, ortho-dichlorobenzene provided a good dispersion and a quick evaporation, making it suitable for this study. Fig. 2 shows a low magnification image of pristine SWCNTs dispersed in ortho-dichlorobenzene and subsequently dropcasted on top of a continuous carbon film grid. In other solvents we realized that either aggregates predominated over individual nanotubes (hexane, acetonitrile) or the solvent would leave a residue on top of the support making it hard to visualize individual nanotubes (chloroform, water with 1% pluronic). By SEM analysis it is possible to easily discern between individual SWCNTs, bundles and aggregates.

To optimize the imaging conditions an equilibrium had to be found in terms of resolution and surface sensitivity. With an increasing accelerating voltage, the resolution increases but the surface sensitivity decreases. SWCNTs are small objects with diameters in range of few nanometers and thus high surface sensitivity is needed for the best contrast conditions in SEM. Hence, the best imaging conditions were found for 2 kV electron beam when using TLD detector, which provides the best resolution of all of the tested detectors in FEI Magellan 400 L HRSEM microscope.

### Optimized procedure to allow a precise length measurement by SEM

**Fig. 3:**
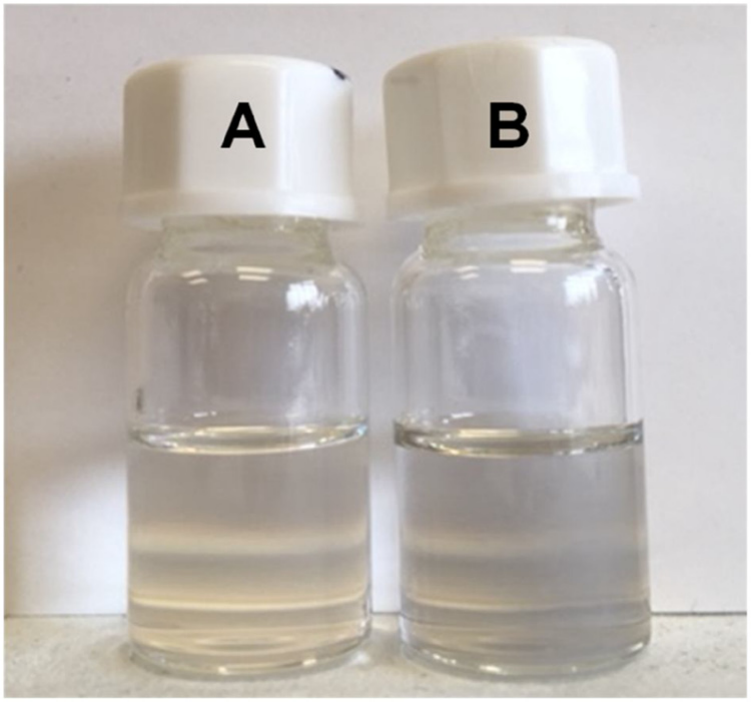
Picture of vials containing ortho-dichlorobenzene (vial A) and an optimized dispersion of SWCNTs in ortho-dichlorobenzene (vial B).

We have developed and optimized a protocol in order to avoid the presence of large CNTs aggregates. Samples were dispersed by sonication of a tiny amount of CNTs in 3 mL of ortho-dichlorobenzene for at least 30 min until a homogenous pale grey suspension was obtained (Fig. 3).

Next, the dispersion was dropcasted onto a TEM copper grid coated with a continuous carbon film, and left to dry. SEM micrographs were acquired in SE mode, which permitted the visualization of SWCNTs previously dispersed in ortho-dichlorobenzene. This imaging modality allows to easily discern between bundles and individual CNTs. SEM acquisition was conducted at 2 kV using a TLD to render surface sensitive images. SEM is time-efficient for the analysis of large amount of samples as tens of individual SWCNTs can be measured from every single image. On the other hand, analyses of these same samples by TEM would render low contrast images where SWCNTs would be less visible, since transmitted electrons are used to visualize the sample and the TEM support is also consisting on carbon. In the case of SEM, secondary electrons give a high contrast topological image of the studied material.

### Method validation

**Fig. 4:**
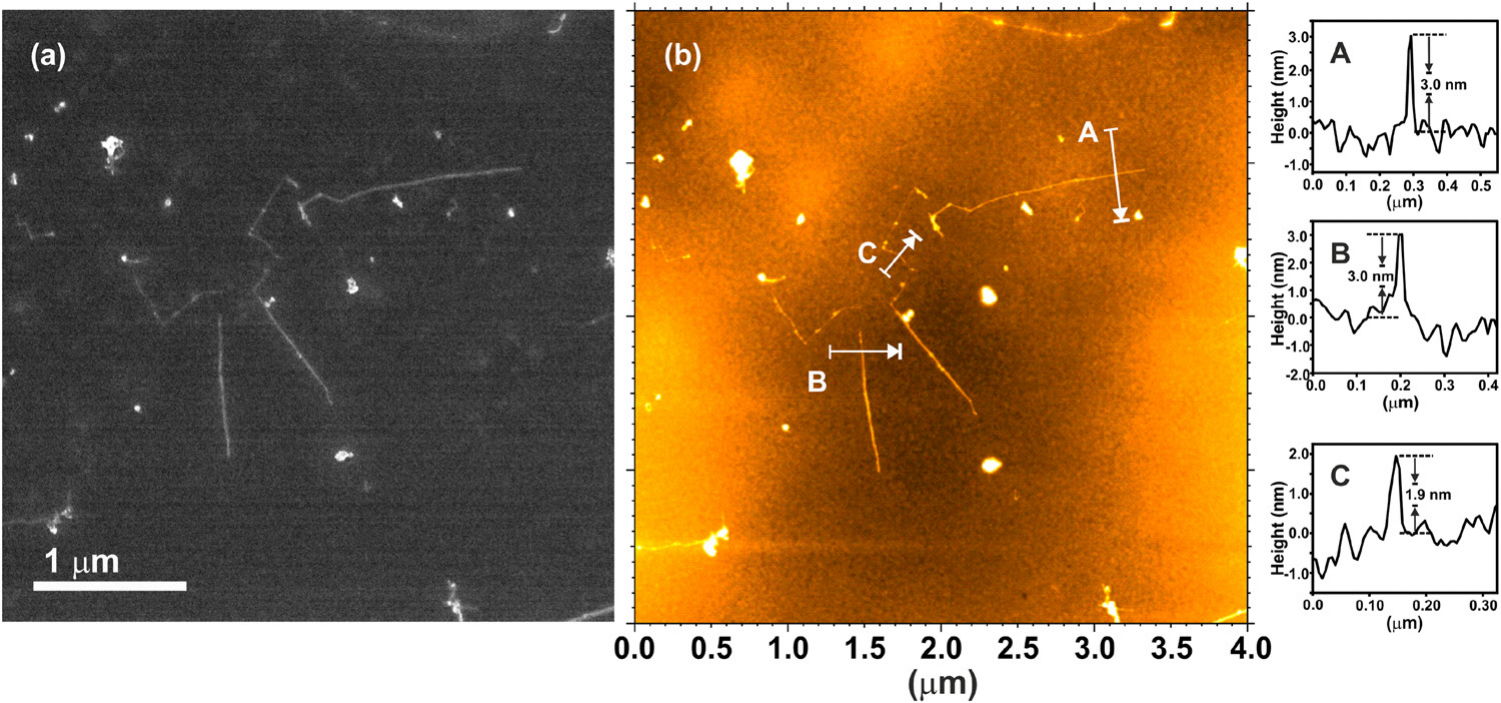
a) SEM and b) AFM images from the same area of SWCNTs (as-received) deposited onto a carbon coated copper grid. Height profiles across several SWCNTs (along the white arrows) are included as insets (indicated with A, B and C).

**Fig. 5:**
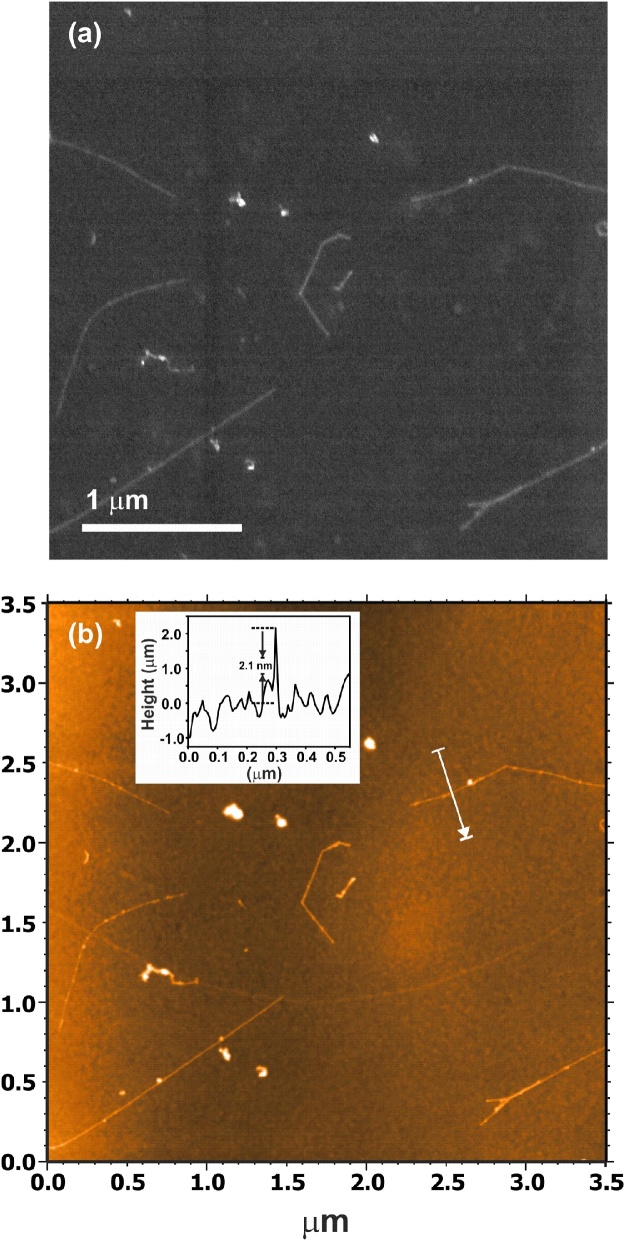
a) SEM and b) AFM images from the same area of SWCNTs (as-received) deposited onto a carbon coated copper grid. A height profile recorded along the white arrow is included as an inset.

Since AFM is the technique most frequently used to determine the length of SWCNTs, we have imaged the same area of SWCNTs deposited on a carbon coated Cu grid and previously imaged by SEM. Fig. 4, Fig. 5 show SEM and AFM images of two different areas. As observed, both techniques allow to easily detect the SWCNTs and could be used to determine their length with the same precision. We have also confirmed by AFM that the observed nanotubes are indeed individual objects. The recorded height profiles across different nanotubes, included in Fig. 4, Fig. 5, agree with the values reported for SWCNTs (with no bundles). The average diameter of the carbon nanotubes in the as-received material is 2.1 nm (value provided by the supplier). Moreover, SEM presents some advantages with respect to AFM for the purpose of measuring length of SWCNTs: (i) imaging of a given area is faster in SEM than AFM, and (ii) the acquisition of low magnification images by AFM is limited. In some AFM equipments, an optical microscope is coupled as an accessory for the latter purpose, but does not have the necessary resolution to detect SWCNTs.

## Experimental

Elicarb^®^ chemical vapor deposition (CVD) grown SWCNTs were provided by Thomas Swan Co. Ltd. The sample also contains a fraction of double-walled CNTs. Steam treatment was performed following a previously reported protocol [25]. Ortho-dichlorobenzene (C_6_H_4_Cl_2_) was purchased from Sigma-Aldrich (99% purity). To perform the analysis of the samples, SWCNTs were initially sonicated during at least 30 min in different solvents till good dispersion was achieved. Then, the so-obtained lightly grayish suspension was drop casted onto various supports. SEM was performed on a FEI Magellan 400 L XHR. The use of TLD at a landing energy of 2 kV enabled to obtain surface sensitive images with spatial resolution below 1 nm, allowing the visualization of individual CNTs. Acquired images were analyzed using Digital Micrograph software. Atomic Force Microscopy (AFM) images were acquired with an Agilent 5100. A tapping mode was employed using FORT tips, with a frequency of 65 kHz and a force constant of 3 N/m.

## Conclusions

We have optimized the conditions necessary to measure the length of SWCNTs by means of SEM. As a result of the investigation, ortho-dichlorobenzene was found to be the best solvent for successful dispersion of CNTs, while continuous carbon film supported on TEM Cu grid was chosen as the best support for the deposition and visualization by SEM. Recorded images were taken at 2 kV of accelerating voltage and SE mode using TLD detector were selected as the best imaging conditions.
